# Nutil: A Pre- and Post-processing Toolbox for Histological Rodent Brain Section Images

**DOI:** 10.3389/fninf.2020.00037

**Published:** 2020-08-21

**Authors:** Nicolaas E. Groeneboom, Sharon C. Yates, Maja A. Puchades, Jan G. Bjaalie

**Affiliations:** Neural Systems Laboratory, Institute of Basic Medical Sciences, University of Oslo, Oslo, Norway

**Keywords:** image processing, rodent atlas, brain, QuickNII, QUINT, workflow

## Abstract

With recent technological advances in microscopy and image acquisition of tissue sections, further developments of tools are required for viewing, transforming, and analyzing the ever-increasing amounts of high-resolution data produced. In the field of neuroscience, histological images of whole rodent brain sections are commonly used for investigating brain connections as well as cellular and molecular organization in the normal and diseased brain, but present a problem for the typical neuroscientist with no or limited programming experience in terms of the pre- and post-processing steps needed for analysis. To meet this need we have designed *Nutil*, an open access and stand-alone executable software that enables automated transformations, post-processing, and analyses of 2D section images using multi-core processing (OpenMP). The software is written in C++ for efficiency, and provides the user with a clean and easy graphical user interface for specifying the input and output parameters. *Nutil* currently contains four separate tools: (1) A transformation toolchain named “Transform” that allows for rotation, mirroring and scaling, resizing, and renaming of very large tiled tiff images. (2) “TiffCreator” enables the generation of tiled TIFF images from other image formats such as PNG and JPEG. (3) A “Resize” tool completes the preprocessing toolset and allows downscaling of PNG and JPEG images with output in PNG format. (4) The fourth tool is a post-processing method called “Quantifier” that enables the quantification of segmented objects in the context of regions defined by brain atlas maps generated with the *QuickNII* software based on a 3D reference atlas (mouse or rat). The output consists of a set of report files, point cloud coordinate files for visualization in reference atlas space, and reference atlas images superimposed with color-coded objects. The *Nutil* software is made available by the Human Brain Project (https://www.humanbrainproject.eu) at https://www.nitrc.org/projects/nutil/.

## Introduction

The process of changing data from one “raw” format to another to make the data suitable for analysis – often referred to as data wrangling or data transformation – is generally required when employing standardized analytical pipelines. A common example is the pre-processing of magnetic resonance data, involving a set of operations to “clean” the raw images before they can be analyzed and interpreted. In basic neuroscience, two-dimensional (2D) images depicting rodent histological brain sections are typical output of animal model studies designed to investigate brain structure, function, and disease. Large series of these images, generated with a broad repertoire of experimental techniques, serve as the starting point for quantification and mapping of features such as the distribution of molecules, presence of particular cell types, or connections between anatomical regions. The images commonly require transformation prior to analysis, but due to the sheer size and number of image files, transformations are difficult to execute and replicate for biological researchers with limited coding ability. Typically, researchers need access to technology packages such as *Adobe Photoshop*^TM^, *GIMP*^1^, or *NIH ImageJ* (Schneider et al., 2012), but these all have their own limitations such as file size restrictions or lack of parallel processing support. Some basic scripting ability and access to data analysis software such as *ImageMagick* (The ImageMagick Development Team, 2020), *R* (Chambers, 2008) or *Matlab* (MATLAB, 2010) are useful. However, even with these tools and skills, transformations can be time consuming when applied to hundreds of images for whole brain comparative studies.

**FIGURE 1:**
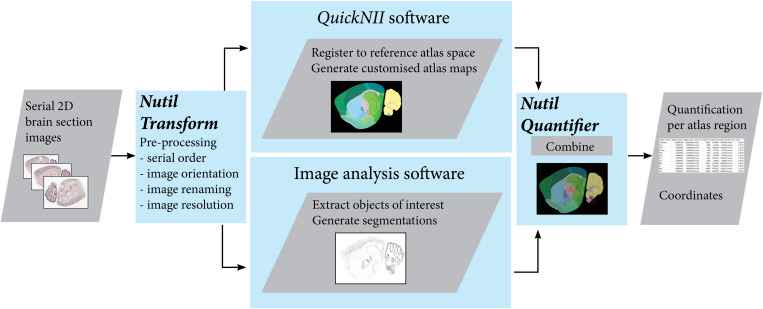
The QUINT workflow utilizes three software tools to extract, quantify and spatially analyze labeled features in series of images of histological mouse or rat brain sections. Initially, the *Nutil* Transform operation is used to correct the orientation and serial order of the images, to rename the files, and to downscale the images. The images are then registered to a reference atlas with *QuickNII*, and segmented with an image analysis software such as *ilastik*. The atlas maps and segmentations are post-processed with *Nutil* Quantifier to extract quantifications per atlas region and coordinates for visualization in reference atlas space. Image Credit for 2D section images: Allen Institute.

With image pre-processing required for most analytic pipelines (Yates et al., 2019), there is a need for access to user-friendly tools that can perform the most commonly required transformations. Furthermore, for brain microscopy data, researchers typically endeavor to spatially analyze features in the images by sorting outputs according to anatomical brain region. In light of published 3D reference atlases for mouse and rat brains (Lein et al., 2007; Oh et al., 2014; Papp et al., 2014; Kjonigsen et al., 2015), and software for generating brain atlas maps that are customized to match the proportions and cutting plane of the sections (Puchades et al., 2019), we have created a pre- and post-processing toolbox for histological brain section images that aims to meet both these needs. An early beta version of the tool was an integral part of the QUINT workflow for quantification and spatial analysis of labeling in rodent brain sections (Yates et al., 2019), see https://www.youtube.com/watch?v=yPkAbSfla_c. Nutil has since been expanded and improved considerably with the present manuscript describing the full range of Nutil functionalities. Based on feedback from our users, we have implemented a proper graphical user interface (GUI) for entering analysis parameters and for selecting different options like i.e., object splitting or customized atlas regions. Nutil can also be used independently of the QUINT workflow to preprocess images in preparation for other downstream processes. It enables automated transformations such as rotation and scaling, cropping, resizing, and renaming; in addition to analytical post-processing of segmentations that are generated from the brain section images, based on input from customized reference atlas maps (Figure 1). *Nutil* is designed specifically for operations on very large images and is therefore optimized for speed, memory usage and parallelization. The toolbox is intended for use in combination with one of the open source image analysis tools that are currently available, e.g., *NIH ImageJ* (Schneider et al., 2012) or ilastik (Berg et al., 2019).

## Overview of Functionality

*Nutil* aims to both simplify and streamline the mechanism of pre-and-post processing 2D brain image data. *Nutil* is developed as a stand-alone application for Windows with a GUI requiring little-to-no experience to execute. The user specifies the input and output parameters for the operations in a template in the GUI, which is saved as a text file (in .NUT format) prior to initiating the operation. Pre-processing operations include 2D transformations of extremely large tiled TIFF files (rotation, flipping, and scaling), in addition to renaming, copying, and downsizing (Transform). The tiled TIFF format was selected for the Transform operation as it allows for optimal memory usage, as well as being an output file type of microscope scanners. Some users may need to convert their images to tiled TIFF format to enable transformation with *Nutil* Transform, and so an image format converter (JPEG/PNG to tiled TIFF format) is included in the *Nutil* software (TiffCreator). To complete the set, *Nutil* also includes a resizing operation (Resize) that enables downscaling of PNG and JPEG images to PNG format. Post-processing with *Nutil* (Quantifier) is based on analysis of segmented images in the context of brain regions defined by a 3D reference atlas such as the Allen Adult Mouse brain reference atlas (Lein et al., 2007; Oh et al., 2014) or the Waxholm Space Atlas of the Sprague Dawley rat brain (Papp et al., 2014; Kjonigsen et al., 2015; Osen et al., 2019). All functions operate in batch mode, and operate in parallel on multiple CPUs.

### Transform

The *Nutil* Transform function is seemingly straight-forward, since in reality it only applies a generic 2D TSR (Translation, Scaling, and Rotation) matrix to an image. However, due to the sheer size of the image files to be transformed, *Nutil* has been designed with several optimization methods to prevent memory problems and to increase execution speed. For example, a single image of size 70,000 × 50,000 pixels takes up almost 10 GB of memory, with a need for additional copies to be stored in memory during rotation. Even worse, if the user were to perform eight transformations on multiple cores, the amount of RAM required would exceed 160 GB.

In order to solve the memory problem, *Nutil* Transform (rotate, flipping, and renaming) operates on tiled TIFF files only, with a separate TiffCreator tool included for converting JPEG/PNG images to the required tiled TIFF format. Tiled TIFF files are effectively compressed (JPEG/LZW) folders of smaller-sized images (tiles) that collectively make up the entire image. By operating (loading/releasing) on these tiles instead of an entire image, memory consumption is greatly reduced; however, this comes at the expense of increased code complexity, with a requirement to keep track of/sort stacks of recently used tiles. To enhance execution speed, *Nutil* is written in C++, which is highly optimized for low-level execution of memory-intensive parallel code.

Nutil performs two kinds of downsampling of TIFF images: original scanner images that are large (usually around 30,000 × 18,000 and up to 80,000 × 70,000 pixels) to about 1–25% of the original area, and original images to very small thumbnails. Nutil does not currently support any form of antialiasing when downsampling TIFF images, only pointwise sampling. In its most basic form, single pixels are selected as representative for the area. Supplementary File 1 summarizes Nutil operating times for image transformations in batch mode on several platforms.

#### Pseudocode Block

1. Read input list of files with their corresponding 2D transform parameters.
2. For each input file, verify that parameters are correct and image files exist.
3. For each file (in separate threads) do:
  (a) Open the input TIFF file, verify that it is indeed tiled.
  (b) Calculate new file resolution and boundaries – rotating an image will alter the resolution.
  (c) Perform 2D transformation on each separate tile, keep an optimized list of recently accessed tiles for speed improvements. Also, simultaneously calculate new bounding boxes for the rotated image (auto-cropping step).
  (d) Save the rotated image to a temporary file, close file handle.
  (e) Create a new file with auto-cropped boundaries calculated in (c) and start copying from the temporary file to the final image.
  (f) If the thumbnails creation option is toggled, open the finished auto-cropped image and downsize it. Save as a.png (or.jpg) in a separate thumbnail folder. No anti-aliasing step is performed here.

### TiffCreator

Even though the tiled TIFF format is useful, very few image services exist that are able to efficiently convert images to tiled TIFF format. Standard image software such as *GIMP* and *Adobe Photoshop* are certainly able to load tiled TIFFs (that are not too large), but are unable to save them. The conversion tool in *ImageMagick* can successfully create images, but require the knowledge of command line parameters in addition to being slow and non-parallelized. The *Nutil* TiffCreator function solves this problem by producing tiled TIFF files from JPEG or PNG images, and employs the support of multiple CPUs for efficient, parallelized operations. TiffCreator lets the user specify the input directory containing JPEG/PNG files and the output directory, in addition to some TIFF-specific parameters (size of sub-tiles, etc.).

#### Pseudocode Block

1. Read input directory and create a list of all image files.
2. For each file in the list
  (a) Load the image
  (b) Convert to a tiled TIFF format
  (c) Save the tiled TIFF

### Resize

The *Nutil* Resize operation is a helpful post processing operation that allows the user to downscale PNG and JPEG files in bulk. An input directory, output directory, compression type and resize factor are specified in the GUI, and the program automatically identifies and transforms all files from the source directory to the target directory. The resize factor can be either a fixed-width number or a percentage of the original size. Nutil does not currently support enlargement of images as this serves no purpose in the context of histological image analysis. As with the other operations in *Nutil*, Resize operates in parallel and utilizes all (or as many as specified) cores on the system it is running.

#### Pseudocode Block

For each file in the list

1. Open the file
2. Resize the file (using either bilinear filtering or no filtering)
3. Save the file

### Quantifier

Quantifier is a post processing operation for the extraction, quantification and spatial analysis of labeling in 2D rodent brain section images (for example, immunohistochemical labeling). Nutil contains all the necessary label files for analysis of features in the following atlases: Allen brain atlas Common Coordinate Framework v3, 2015 and 2017 versions (^®^2004 Allen Institute for Brain Science. Allen Mouse Brain Atlas. Available from: http://download.alleninstitute.org/informatics-archive/current-release/mouse_ccf/annotation/) (Lein et al., 2007; Oh et al., 2014) and the Waxholm Space Atlas of the Sprague Dawley rat brain v2 and v3 (Available from: https://www.nitrc.org/projects/whs-sd-atlas) (Papp et al., 2014; Kjonigsen et al., 2015; Osen et al., 2019). To run Quantifier, *Nutil* requires segmentations that are generated from the brain section images, with the labeling of interest displayed in a unique RGB color (Red Green Blue color model) and reference atlas maps customized to match the proportions and cutting plane of the sections (generated with the QuickNII software, available from: https://www.nitrc.org/projects/quicknii) (Puchades et al., 2019). Quantifications may be performed on the entire image or in regions defined by masks. *Nutil* quantifies labeling in each parcellated brain region, extracts spatial coordinates for visualization in 3D reference atlas space, and generates output including: reports (in CSV or HTML format), coordinates (in JSON format), and reference atlas map images superimposed with the extracted features that are color-coded according to their assigned anatomical location (Yates et al., 2019).

Quantifier starts by applying a breadth-first search (BFS) method to identify areas of interest in a series of images as specified by colors defined in the *Nutil* GUI. This method proceeds by separating foreground areas (of interest) from background areas, before calculating various statistical measures (area, centroid, shape). When this process is complete, Quantifier continues by anchoring each identified object to a specific brain region by looking up the individual pixel coordinates in the anchoring map provided by the *QuickNII* output data. If an area overlaps N regions of the brain, the area is either assigned to one of the regions at random (with object splitting switched OFF) or split into N distinct areas with IDs coupled to their respective regions (with object splitting switched ON). In addition, various filters can be applied such as minimum and maximum object size, which are useful for removing noise. Functionality tailored specifically for connectivity data has been implemented, with support for masks to enable differentiation of connections in the right and left hemispheres. The object splitting feature was also implemented for connectivity data as connections typically span multiple atlas regions. With object splitting switched ON each object pixel is registered to its respective atlas region. However, users will typically want to switch this feature OFF for small objects such as cells, to enable accurate counting. Object splitting invalidates the object counts as objects are split at region boundaries.

The output results are assembled based on information provided in the *Nutil* GUI, which includes the option to define customized regions (ensembles of reference brain region IDs) together with a name (“Cortex”) and color (“red”) (via an Excel template; see Supplementary File 2). This enables the user to constrain the output data to specific areas of the brain (e.g., “Amygdala,”, “Hippocampus,” “Primary Somatosensory Cortex”). Report outputs are written as a series of files in CSV or HTML format containing report information per section, and for all sections combined (global information), and are organized first by all the regions listed in the relevant reference atlas (e.g., Allen adult mouse brain reference atlas) and second by the optional user-defined custom regions. Moreover, *Nutil* generates 3D point clouds, colorized based on report specifications that may be visualized in the online *MeshView* atlas viewer^2^. The point clouds are exported in a simple JSON-formatted text file that is compatible with Python, containing a raw list of 3D coordinates collected together with color-coding based on the input report. Support for specifying the required point cloud density has been implemented to enable the extraction of coordinates for datasets with very large objects (such as those typical of connectivity data, where a user may, for example, request extraction of one coordinate per 5th object pixel).

#### Pseudocode Block

1. For each input file, verify that parameters are correct and that the files in the file list exist.
2. Create output directories.
3. For each slice (in separate threads) do:
4. Open segmented image.
5. Apply masks, calculate some statistics.
6. Perform a breadth-first algorithm that counts the number of areas, and place them into an object list.
7. Open the corresponding atlas slice and assign each area to a unique atlas ID.
8. Calculate various area statistics.
9. Create a visual.png file by merging the segmented image file with the atlas image file, and add label text and colors to the identified areas.
10. When all threads have completed, combine areas into one object and calculate both individual slice and global statistics.
11. Write reports:
12. Individual slice reports.
13. Combined area reports.
14. Write 3D data (perform an inverse matrix operation to get back to atlas space).
15. Clean up memory and temporary files.

## Technical Specifications

*Nutil* is written in C++ using standard Qt^3^ libraries, and is optimized for parallel operations on multiple CPUs. *Nutil* is downloaded as a zip archive file and can be extracted and run anywhere on the computer. No installation executable is necessary, and the directory can be moved around the file system as required. Settings data are stored in the local program folder. Nutil does not currently utilize any GPU extensions. The external libraries that are used in *Nutil* are:

1. Libtiff for fast and efficient TIFF file handling^4^
2. LibXLNT for Excel file I/O^5^

### Hosting and Updates

*Nutil* is available from two locations:

1. Windows binaries (no installer required): https://www.nitrc.org/projects/nutil/
2. Source code: https://github.com/leuat/nutil/

### Hardware Requirements

*Nutil* is a stand-alone program that can be executed on either a server, desktop or laptop computer. *Nutil* will employ all the cores that are available to your current system. While there are no specific hardware limitations, the processing time is dependent on the system’s compute power. The more cores and memory you have available, the faster the operations will be performed. However, running *Nutil* on a single-core laptop is also possible. This way, we allow the user to not be constrained by any hardware restrictions, as Nutil should be able to run on almost any computer.

## Use Cases

### Use Case Material

Three mouse brain image series were exported from the Allen Brain Atlas Data Portal with a Python script. The images were exported in JPEG format, and served as the test series for the TiffCreator, Transform, and Quantifier functions. The first series (use case A) included 20 sagittal mouse brain sections from the left hemisphere labeled for parvalbumin by *in situ* hybridization, and may be viewed here: http://mouse.brain-map.org/experiment/show/75457579 (Specimen 06-0419, Probe RP_060523_03_E07; ^®^2004 Allen Institute for Brain Science). The second *in situ* hybridization series (use case B) include 57 coronal sections labeled for parvalbumin^6^ (Specimen 335-1125, Probe 071204_01_E06; ^®^2004 Allen Institute for Brain Science. Allen Mouse Brain Atlas. Available from: mouse.brain-map.org) (Lein et al., 2007; Oh et al., 2014). The third series (use case C) was a connectivity dataset displaying projections from the primary somatosensory mouth area. Nine images were selected for the use case and can be viewed here: https://connectivity.brain-map.org/projection/experiment/112936582 (Mouse strain: C57BL/6J, Tracer type: EGFP; ^®^2011 Allen Institute for Brain Science. Allen Mouse Brain Connectivity Atlas).

### TiffCreator and Transform

**FIGURE 2:**
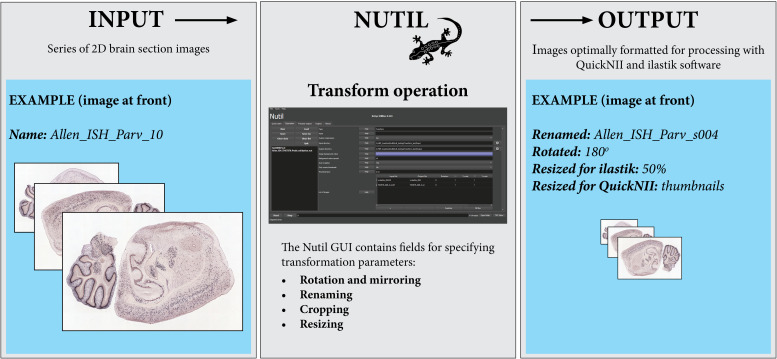
The Nutil Transform operation takes series of images as input, and outputs copies of the images that have been formatted based on parameters defined by the user in the *Nutil* GUI. Nutil Transform includes the option to rotate and mirror, rename, crop, resize, and convert file type. Image Credit for 2D section images: Allen Institute.

TiffCreator enables batch conversion of JPEG/PNG images to the tiled TIFF format that is compatible with the Transform function. Transform then allows the user to perform a variety of image pre-processing steps such as scaling, rotation, and renaming, which are prerequisites for analysis by the method described in the other use cases (illustrated in Figure 2), but also for analysis by other means. For both TiffCreator and Transform, all the input and output parameters were entered in the *Nutil* GUI, and the desired operation run in batch mode. As *Nutil* Transform operates on tiled TIFF images only, the first operation for all the use cases was to convert the JPEG images to tiled TIFF using TiffCreator. Next, Transform was used to rename the files – to make them compatible with *QuickNII* and *Nutil* Quantifier – and to create the thumbnails required for registration to reference atlas space. As the images were well-organized and small in size, conversion from tiled TIFF to PNG was the only additional step required before segmentation with *ilastik* (Berg et al., 2019).

Note that all *Nutil* Transform operations – such as rotation, scaling and thumbnail creation – may be implemented in one process or separately. The thumbnail feature is particularly useful for visualizing the result of a transformation without having to open large images, as it allows the application of a resize factor with image export in PNG format, in addition to export of the original size transformed images. For rotation, the user may choose any angle and define the background color (i.e., black or white) in the new image. The rotation feature includes an auto-cropping function, which ensures the removal of redundant pixels. The *Nutil* GUI has an inbuilt user manual, accessible via help buttons. A user manual is also part of the *Nutil* software package and will be updated with new releases.

### Quantifier: *In situ* Hybridization Dataset (Use Cases A and B)

**FIGURE 3:**
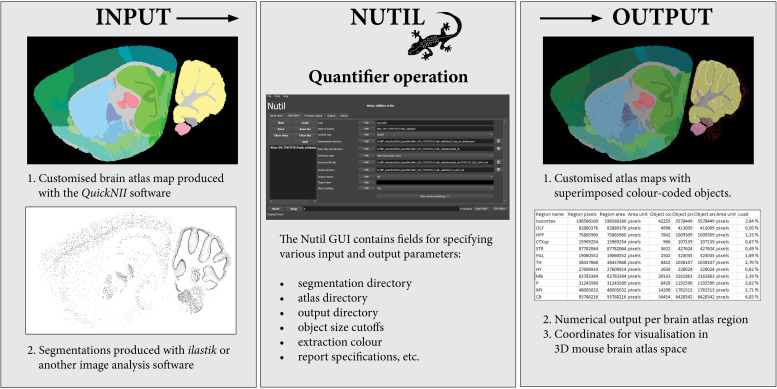
The *Nutil* Quantifier operation takes atlas maps and segmentations as input, and outputs reports with quantifications per atlas region, atlas maps with superimposed color-coded objects and point clouds for visualization in reference atlas space. The *Nutil* GUI supports specification of parameters such as color for extraction, minimum and maximum object size, and coordinate file requirements. The GUI includes an in build user manual accessible via help buttons.

Quantifier allows the quantification and spatial analysis of labeling in series of mouse or rat brain section images based on input from segmentations and customized atlas maps, both generated from the section images (Figure 3). Quantifier was used to extract, quantify and assign anatomical location to parvalbumin positive cells that were extracted from the image series that is described in section “Use Case Material”. The images were pre-processed with *Nutil* TiffCreator and Transform as described above. For the sagittal parvalbumin example, derived input files (customized atlas maps and segmentations) and output files (Csv reports, point cloud, and atlas map images with superimposed features) are available for download from the EBRAINS KnowledgeGraph database ( doi: 10.25493/6DYS-M3W)^7^.

**FIGURE 4:**
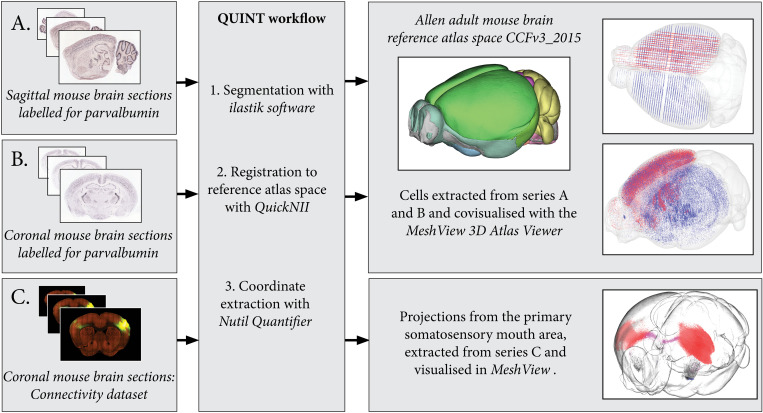
By processing several image series with the QUINT workflow, point clouds representing labeled features can be covisualized in reference atlas space allowing comparisons of expression patterns. Here, parvalbumin positive cells are extracted from two mouse brain section series: one cut in the sagittal plane **(A)** and the other in the coronal plane **(B)**. The cells are covisualized in Allen Adult Mouse Brain Reference atlas space with the *MeshView* atlas viewer. Likewise, the connectivity use case **(C)** displays projections from the primary somatosensory mouth area, labeling in all brain areas is quantified and visualized in 3D with differential color coding dependent on the brain region (neocortex, red; corpus callosum, pink; striatum, black). Image Credit for 2D section images: Allen Institute. Series: http://mouse.brain-map.org/experiment/show/75457579, http://mouse.brain-map.org/experiment/show/79556738, https://connectivity.brain-map.org/projection/experiment/112936582.

To generate the atlas maps, the mouse brain section images were registered to Allen Adult Mouse Brain Reference Atlas space (Common Coordinate Framework version 3, 2015) with the *QuickNII* software version 2.1 (Available from: https://www.nitrc.org/projects/quicknii) (Figure 3, Input 1). In a parallel procedure, the section images were classified with the Pixel Classification workflow in the *ilastik* software to identify the parvalbumin positive cells (version 1.3.2rc1; available from: https://www.ilastik.org). Segmentations were generated in PNG format, with RGB color value (0, 0, 0) (black) applied to the class representing the positive cells (Figure 3, Input 2). The Quantifier template in the *Nutil* GUI was populated with the input and output directories, as well as parameters such as minimum and maximum object size and coordinate file requirements. Object splitting was switched OFF. Quantifier was subsequently applied to all the images in batch mode. The point cloud was visualized with the *MeshView* Atlas Viewer (AMBA version 3 2015, available from: https://www.nitrc.org/projects/meshview). The same procedure was repeated for use case B, and the datasets were co-visualized in 3D (Figure 4). The processing of the dataset with Nutil Quantifier on a standard laptop with 2 to 4 cores and 16 GB of memory took less than 10 min, including selection of the files and analysis parameters in the GUI interface.

### Quantifier: Connectivity Dataset (Use Case C)

The connectivity dataset was preprocessed with Nutil Transform, segmented with *ilastik* and registered to Allen Adult Mouse Brain Reference Atlas space (Common Coordinate Framework version 3, 2015) with *QuickNII* in the same way as described for the *in situ* hybridization datasets. The dataset was processed with Nutil Quantifier with object splitting switched ON, with masks to differentiate connections in the right and left hemisphere and with a reduced point cloud density to enable visualization in 3D (Figure 4).

### Quantifier Validation

To validate the use case output, we compared the results obtained with *Nutil* Quantifier for the *in situ* hybridization dataset with the raw expression values provided by the Allen Brain Institute for the same dataset and brain regions. Despite considerable differences in quantification methods (Bohland et al., 2010) and minor differences in the atlas registration, the outputs were comparable as demonstrated in Supplementary File 3.

A proper validation of *Nutil* Quantifier was done with a synthetic dataset of two sections, each with a set number of objects of known size (in pixels) and anatomical location. This dataset confirms that the quantification data delivered by *Nutil* Quantifier are correct (Supplementary File 4). Further validation is provided in Yates et al. (2019).

## Discussion

With the development of microscopes and scanners for whole section imaging and volumetric data acquisition, scientists are able to collect high-resolution images of different organs including the brain. The sheer size of these images has necessitated the development of new software tools for viewing, transforming and analyzing imaging data; but are typically only available commercially, and often with restrictions in terms of functionality that do not promote optimal analysis. For human brains, methods for 2D image reconstruction from multiple blocks have allowed researchers to conduct multimodal imaging studies (Hashimoto et al., 2016). There are also some open-access tools such as “HistoStitcher” (Chappelow et al., 2011) that enable specific pre-processing operations like reassembly of tissue fragments. Most pre-processing steps may be done with scripts, many available online; however, for a neuroscientist with limited programming experience, pre-processing can be a slow and painful process. There is a need for open-access tools that operate without the need for coding expertise with functionality tailored specifically for series of histological brain section images.

With *Nutil*, neuroscientists will find a user-friendly and convenient tool, specifically designed with serial rodent brain sections in mind. It enables image transformations in an automated batch operation, making downstream image analysis accessible to the wider community. In a separate post-processing operation, *Nutil* allows the quantification and spatial analysis of labeling in 2D image series of rodent brain sections, when used in combination with *QuickNII* (Puchades et al., 2019) – an open-access software for registering section images to reference atlases – in addition to an image segmentation tool such as *NIH ImageJ* (Schneider et al., 2012) or *ilastik* (Berg et al., 2019). Several groups have developed custom codes and workflows along the same lines, for the analysis of whole brain volumes by registration to reference atlases. In a recent study, Kim et al. (2017) used the 3D Allen mouse reference atlas (Lein et al., 2007; Oh et al., 2014) for quantification of several interneuronal types in serial two-photon tomography data. By registering all datasets to the same reference space, they were able to discover sex differences in specific atlas sub-regions. Other studies rely on block-face photography (Vandenberghe et al., 2016) or 3D reconstruction (Majka et al., 2012). Likewise, *Nutil* has the potential to contribute to discoveries based on histological experimental data without the need for 3D volumes or 3D reconstruction. Several other groups have proposed tools for analysis of 2D mouse brain section images, without the need for 3D reconstruction (Furth et al., 2018; Xiong et al., 2018); however, the requirement for coding knowledge restricts the accessibility of these methods. The AIDAhisto tool enable atlas based quantifications but lack multi-angle adjustment (Pallast et al., 2019).

In contrast to the cited studies, *Nutil* Quantifier may be applied to both mouse and rat brain data, as both mouse and rat brain reference atlases are inbuilt in the *Nutil* software, with *QuickNII* versions available for both species. *Nutil* Quantifier supports quantification of both small objects such as cells, typical of immunohistochemistry and *in situ* hybridization data, and the exploration of large objects that span multiple atlas regions, such as those typical of connectivity data. There is also a feature for combining masks with the atlas maps, to allow comparative analysis of right and left hemisphere expression. As the TiffCreator, Transform, Resize, and Quantifier operations run independently, TiffCreator, Transform, and Resize may be used to process any type of image, and can be incorporated into workflows that do not include the Quantifier operation. However, the pre-processing package is ideally suited for use with Quantifier; with all operations running on multiple CPUs, which drastically increases processing speed, making them ideal for batch processing of section images. By combining information from segmented images with corresponding atlas maps produced with *QuickNII* (Puchades et al., 2019; Yates et al., 2019), several datasets may be analyzed and then compared (Figure 4), offering new analytical possibilities as discussed in depth in Bjerke et al. (2018). The present developments are part of a larger European effort to establish tools and services for neuroscience research (Amunts et al., 2019).

## Data Availability Statement

Publicly available datasets were analyzed in this study. This data can be found here: http://mouse.brain-map.org/experiment/show/75457579, 10.25493/6DYS-M3W.

## Author Contributions

NG created the *Nutil* software, contributed to the writing of the technical parts of the manuscript, and to the design of the validation studies. SY performed *Nutil* analyses, performed the validation studies, contributed to the development of the *Nutil* software, and contributed to writing the manuscript. MP conceived the study, supervised the analysis and the development of *Nutil*, and wrote the manuscript. JB conceived the study, supervised development of software tools, contributed with infrastructure, and contributed to writing of the manuscript. All authors contributed to the article and approved the submitted version.

## Conflict of Interest

The authors declare that the research was conducted in the absence of any commercial or financial relationships that could be construed as a potential conflict of interest.

## Footnotes

1 www.gimp.org

2 https://www.nitrc.org/projects/meshview

3 https://www.qt.io/

4 http://www.libtiff.org/

5 https://github.com/tfussell/xlnt/

6 http://mouse.brain-map.org/experiment/show/79556738

7 https://kg.ebrains.eu/search/instances/Dataset/8e8ac473-08fc-4510-bf81-e6d94c9c15d6
