# Supplementary material for: Nutil: A Pre- and Post-processing Toolbox for Histological Rodent Brain Section Images
Source: Front Neuroinform. 2020 Aug 21;14:37. doi: 10.3389/fninf.2020.00037 (PMC7472695; doi:10.3389/fninf.2020.00037)
Supplement: Supplementary file 1 [file Data_Sheet_1.PDF]

Supplementary File 1.

Summary of Nutil operating times for image transformations in batch mode on several platforms

Transformations were performed on 16 images of size: 24724 x 16558 pixels and 600 MB. The transformation parameters were as follows: 45 degree rotation, flipping on the x-axis and renaming of all files.

**Computer**	**Processor**	**No. of threads used**	**Operating system**	**Memory**	**Operating time**
Standard Laptop	Intel(R) Core(TM) i7-4712MQ CPU @2.30Hz	8	64-bit	16 GB	22 min 7 sec
Standard Desktop	Intel(R) Core(TM) i7-6700 CPU @3.40GHz	8	64-bit	32 GB	9 min 23 sec
Workstation-Z840	Intel(R) Xeon(R) CPU E5-2643 v3 @3.40Ghz	24	64-bit	192 GB	5 min 9 sec
