# Supplementary material for: Nutil: A Pre- and Post-processing Toolbox for Histological Rodent Brain Section Images
Source: Front Neuroinform. 2020 Aug 21;14:37. doi: 10.3389/fninf.2020.00037 (PMC7472695; doi:10.3389/fninf.2020.00037)
Supplement: Supplementary file 2 [file Data_Sheet_2.PDF]

Supplementary File 3.

Validation of the quantifications obtained for the use case ISH_79457579

Quantification of signal with the QUINT workflow show the same expression trend as the raw expression quantifications from Allen database found here: (http://mouse.brain-map.org/experiment/show/75457579) (Specimen 06-0419, Probe RP_060523_03_E07; ©2004 Allen Institute for Brain Science. Allen Mouse Brain Atlas). (Lein et al., 2007; Oh et al., 2014)

Small expression differences may be due to differences in both the atlas registration methods and quantification methods. The raw expression quantifications from Allen Institute takes into account labelling intensity and are described in Bohland et al. 2010 Methods, doi: 10.1016/j.ymeth.2009.09.001.

Custom atlas regions	Parvalbumin load (QUINT)	ISH raw exp (Allen Institute)
IC	2,84 %	7,69
OLF	0,50 %	2,84
HPF	1,33 %	5,14
CTXsp	0,67 %	3,08
STR	0,49 %	3,96
PAL	1,69 %	7,77
TH	2,70 %	6,08
HY	0,82 %	4,43
MB	3,39 %	10,57
P	3,82 %	11,06
MY	3,71 %	9,63
CB	6,85 %	19,16

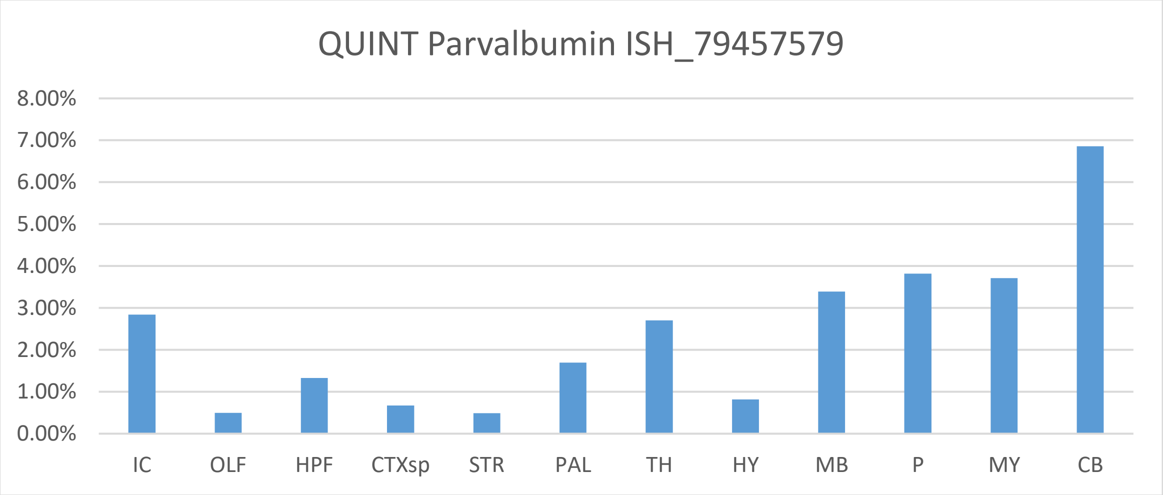

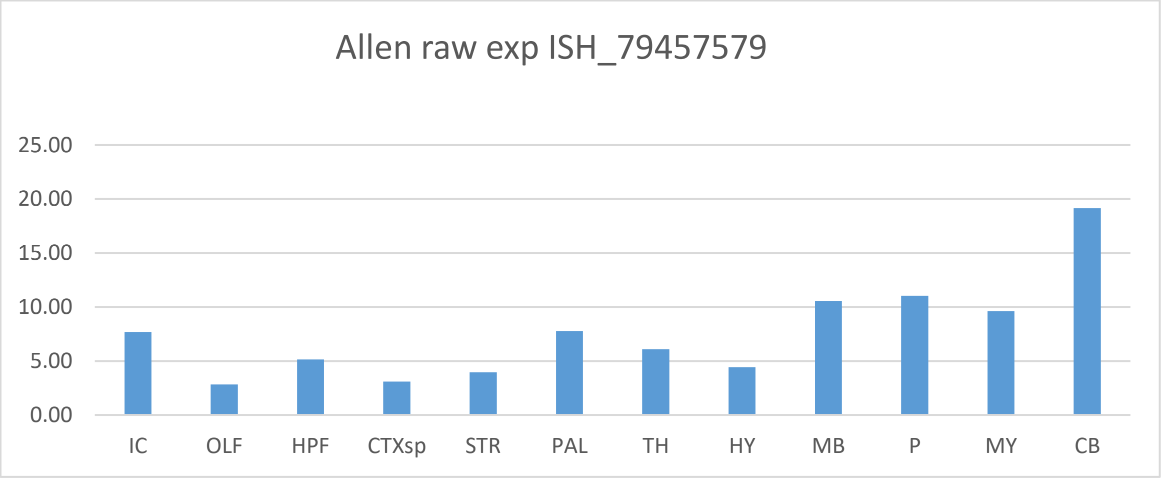
Allen raw exp ISH_79457579
25.00
20.00
15.00
10.00
5.00
0.00
IC
OLF
HPF
CTXsp
STR
PAL
TH
HY
MB
P
MY
CB
