# Supplementary material for: Nutil: A Pre- and Post-processing Toolbox for Histological Rodent Brain Section Images
Source: Front Neuroinform. 2020 Aug 21;14:37. doi: 10.3389/fninf.2020.00037 (PMC7472695; doi:10.3389/fninf.2020.00037)
Supplement: Supplementary file 3 [file Data_Sheet_3.PDF]

Supplementary file 4.

Synthetic validation dataset: Two segmentation (s0218 and s0314) files containing objects with known numbers of pixels were generated and run through the Nutil Quantifier tool. Numerical results show correct calculations and attribution to the right atlas region.

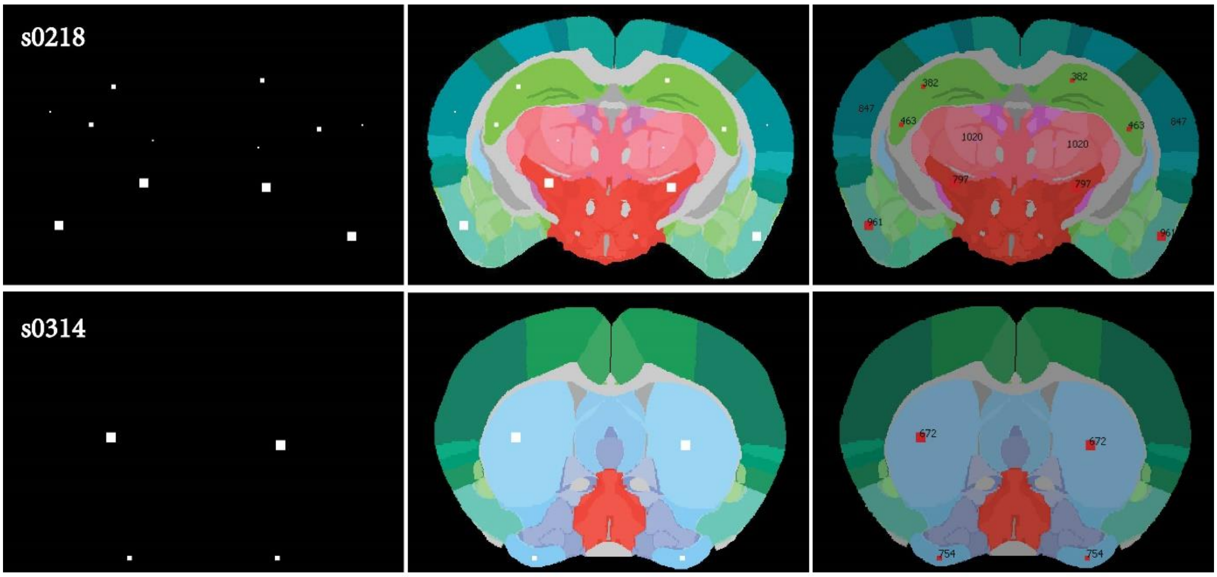

Section ID	Object pixels	Object area	units	Center X	Center Y	Region ID	Region Name
s0218	100	100	pixels	296,50	205,50	797	Zona incerta
s0218	100	100	pixels	158,50	200,50	797	Zona incerta
s0218	100	100	pixels	62,50	248,50	961	Piriform area
s0218	100	100	pixels	392,50	260,50	961	Piriform area
s0218	25	25	pixels	99,00	135,00	463	Field CA3
s0218	25	25	pixels	356,00	140,00	463	Field CA3
s0218	25	25	pixels	124,00	92,00	382	Field CA1
s0218	25	25	pixels	292,00	85,00	382	Field CA1
s0218	4	4	pixels	287,50	160,50	1020	Posterior complex of the thalamus
s0218	4	4	pixels	168,50	152,50	1020	Posterior complex of the thalamus
s0218	4	4	pixels	52,50	120,50	847	Primary auditory area, layer 5
s0218	4	4	pixels	404,50	135,50	847	Primary auditory area, layer 5
s0314	100	100	pixels	292,50	156,50	672	Caudoputamen
s0314	100	100	pixels	113,50	148,50	672	Caudoputamen
s0314	25	25	pixels	289,00	272,00	754	Olfactory tubercle
s0314	25	25	pixels	133,00	272,00	754	Olfactory tubercle
